# Comparative Assessment of the Antibacterial and Antibiofilm Actions of Benzalkonium Chloride, Erythromycin, and L(+)-Lactic Acid against Raw Chicken Meat *Campylobacter* spp. Isolates

**DOI:** 10.3390/antibiotics13030201

**Published:** 2024-02-21

**Authors:** Dimitra Kostoglou, Athina Vass, Efstathios Giaouris

**Affiliations:** Laboratory of Food Microbiology and Hygiene, Department of Food Science and Nutrition, School of the Environment, University of the Aegean, 81400 Myrina, Lemnos, Greece; fnsd21001@fns.aegean.gr (D.K.);

**Keywords:** *Campylobacter* spp., biofilms, benzalkonium chloride, erythromycin, L(+)-lactic acid, antimicrobial action, antibiofilm action, intercellular interactions, food safety, public health

## Abstract

*Campylobacter* spp. are significant zoonotic agents, which cause annually millions of human cases of foodborne gastroenteritis worldwide. Their inclusion in biofilms on abiotic surfaces seems to play a pivotal role in their survival outside of the host, growth, and spread. To successfully mitigate the risks that arise with these bacteria, it is crucial to decrease their prevalence within the food production chain (from farm to the table), alongside the successful treatment of the resulting illness, known as campylobacteriosis. For this, the use of various antimicrobial agents remains actively in the foreground. A general-purpose biocide and cationic surfactant (benzalkonium chloride; BAC), a widely used macrolide antibiotic (erythromycin; ERY), and a naturally occurring organic acid (L(+)-lactic acid; LA) were comparatively evaluated in this work for their potential to inhibit both the planktonic and biofilm growth of 12 selected *Campylobacter* spp. (of which, seven were *C. jejuni* and five were *C. coli*) raw chicken meat isolates, all grown in vitro as monocultures. The inhibitory action of LA was also studied against four mixed-culture *Campylobacter* biofilms (each composed of three different isolates). The results showed that the individual effectiveness of the agents varied significantly depending on the isolate, growth mode (planktonic, biofilm), intercellular interactions (monocultures, mixed cultures), and the growth medium used (with special focus on blood presence). Thus, BAC exhibited minimum inhibitory concentrations (MICs), minimum bactericidal concentrations (MBCs), and minimum biofilm inhibitory concentrations (MBICs) that ranged from 0.5 to 16 μg/mL. Interestingly enough, these values varied widely from 0.25 to 1024 μg/mL for ERY. Concerning LA, the MICs, MBCs, and MBICs varied from 1024 to 4096 μg/mL, with mixed-culture biofilm formation always being more difficult to suppress when compared to biofilm monocultures. In addition, it was evident that intercellular interactions encountered within mixed-culture *Campylobacter* biofilms significantly influenced both the population dynamics and the tolerance of each consortium member to acid exposure. Overall, the findings of this study provide useful information on the comparative effectiveness of three well-known antimicrobial agents for the control of *Campylobacter* spp. under various growth modes (i.e., planktonic, biofilm, monocultures, mixed cultures) that could potentially be encountered in food production and clinical settings.

## 1. Introduction

Campylobacters are the primary causes of reported foodborne gastroenteritis cases in humans in Europe, the USA, and elsewhere [1]. Based on the latest epidemiological data, in Europe in 2022, there were 137,107 confirmed cases of human campylobacteriosis, corresponding to a European Union (EU) notification rate of 43.1 cases per 100,000 population, which is the highest when compared to all the other monitored zoonoses [2]. Thirty-four deaths from campylobacteriosis were reported that year, resulting in an EU case fatality rate of 0.04%. In the USA, campylobacters are estimated to cause annually 1.5 million illnesses [3]. These are microaerophilic, Gram-negative, curved rods that cannot be differentiated into spores, with an optimal growth temperature of 42 °C [4]. As zoonotic organisms, these inhabit the gastrointestinal (GI) tract of avian and mammalian species, usually in symbiotic associations [5]. For instance, *Campylobacter* spp. can be found in high quantities in poultry and other birds, without prompting an immune response. Flocks can indeed be colonized with these bacteria from the age of two weeks, and, once introduced, they spread rapidly throughout the broiler house, likely via the drinking water system, with this spreading also being greatly assisted by the coprophagic behavior of the bird [6,7]. In this way, *Campylobacter* spp. can rapidly reach extremely high numbers in the cecal contents of birds. Hence, levels in the range of 10^5^–10^9^ CFU per g of gut contents are commonly observed, while populations exceeding 10^12^ CFU per g of cecal contents have also occasionally been reported [8,9,10]. This huge commensal colonization enables *Campylobacter* spp. to establish themselves in poultry flocks, which are often the primary source of human infection.

Indeed, epidemiological data, as well as risk assessment studies, indicate that the primary route of campylobacteriosis transmission is the ingestion of contaminated food, principally raw or undercooked chicken meat, or ready-to-eat (RTE) foods, cross-contaminated by raw chicken bacteria, in addition to the ingestion of polluted water or dairy products, commonly unpasteurized milk [11,12]. Poultry meat is also highly vulnerable to contamination during slaughtering and further processing, and, to a certain extent, this is due to the ability of *Campylobacter* spp. to be included in and live through biofilms on a variety of surfaces, usually together with other bacteria (e.g., aerobic pseudomonads), under conditions that might otherwise be quite unfavorable for their survival [13,14,15]. Consequently, if those contaminating bacteria are not neutralized at some later stage, mainly at the industrial level and until leaving the factory, and the meat is not later correctly prepared (through adequate cooking in the kitchen), human infection may occur. This risk is further reinforced when considering that the infectious dose is relatively small, as fewer than 500 organisms are enough to cause the disease [5].

Fortunately, campylobacteriosis is generally self-limited in healthy people, typically subsiding within a week. However, in cases of prolonged and/or severe symptoms, antibiotics may be necessary. The inherent sensitivity of campylobacters to aminoglycosides and macrolide antibiotics (but less so to penicillins) was the reason chemotherapy based on these agents has been suggested as potentially effective since 1977 [16]. Currently, macrolides, including azithromycin and erythromycin (ERY), and fluoroquinolones like ciprofloxacin are the preferred medicines for the treatment of prolonged campylobacteriosis [17]. Regarding ERY, this inhibits the protein synthesis of bacterial cells by reversibly binding with their 50S ribosomal subunit, thus blocking the translocation reaction and the formation of new peptide bonds [18]. However, *Campylobacter* spp., like several other pathogens, have developed multiple mechanisms to resist the pressure generated by antibiotics, oftentimes rendering that treatment ineffective [19]. Not surprisingly therefore, the World Health Organization (WHO) includes campylobacters in the list of the 12 bacteria for which the development of novel antibiotics is urgently needed, mainly due to the rapid increase in fluoroquinolone-resistant strains [20].

The inappropriate use of antibiotics in both humans and animals should account for the great rise in antibiotic resistance in recent years. Additionally, there are growing concerns that the uncontrolled use of other biocides, such as the quaternary ammonium compounds (QACs), which are the active agents of various commonly available disinfectants used in both the food industry and home-care products, may also contribute to this disturbing issue [21,22]. This is because resistance to one antimicrobial agent may sometimes provide cross-protection against another that does not necessarily belong to the same chemical category [23]. Disinfectants are included in biocides that can kill microorganisms [24]. However, if the levels of biocides used are insufficient to completely kill the targeted bacteria, something that may well happen if the latter are included in biofilms, bacterial survival can subsequently lead not only to further adaptation and increased antimicrobial resistance (AMR), but also cross-resistance [25,26,27]. Fortunately, *C. jejuni,* which is the species most associated with human infection, seems to remain generally susceptible to several of the disinfectants used in poultry houses, such as QACs [28]. One common QAC disinfectant is benzalkonium chloride (BAC), which is typically commercialized as a mixture of alkylbenzyldimethylammonium chlorides with alkyl chains ranging from C_8_ to C_18_ in length, with derivatives with carbon chains ranging from C_12_ to C_14_ usually exhibiting greater biocidal activity [29]. The mechanism of the action of QACs, including BAC, involves the perturbation and disintegration of the microbial membrane bilayers by the alkyl chains, and the interruption of the charge distribution of the membrane by the charged nitrogen, ultimately resulting in the leakage of cellular contents [29]. However, although several earlier studies have investigated the potential of BAC against either planktonic growth or the preformed biofilms of various microorganisms, including campylobacters [23,28,30,31,32,33,34], to the best of our knowledge, that action has not yet been evaluated against biofilm formation by those latter bacteria.

In addition to the use of disinfectants, although it sounds impossible to fully eliminate campylobacteria from the food production chain, there are still some other promising physical and chemical strategies that are or could be further used to limit their prevalence at the different stages of this continuity, starting from the farm [35]. For example, during food animal processing, natural organic acids may be used in a sustainable and ecofriendly way to remove pathogens from carcasses and thus decrease their microbial burden [36,37]. It is worth noting that while the acid washing of carcasses is already performed in the USA [38,39], this is not yet applied in Europe, apart from lactic acid (LA), which is used as a beef decontaminant during slaughter [40]. However, organic acids are commonly used as acidifiers in poultry drinking water and as antimicrobial feed additives also [41]. Simultaneously, this acid strategy is assumed to exert a positive impact on the proper functioning of the poultry digestive system [42]. Additionally, the use of LA as a spray for poultry carcasses has often been suggested as an effective means of reducing *C. jejuni* [43]. This acid, like several other organic acids that are used as food preservatives (e.g., acetic, propionic, citric, and benzoic acid), exerts its antimicrobial action mainly through its undissociated molecules which pass freely through the plasma membrane and enter the microbial cell, where they then dissociate, resulting in the reduction of the intracellular pH and the inhibition of the metabolic reactions [44]. However, more recent studies have shown that the effectiveness of LA in reducing *C. jejuni* during the decontamination of poultry carcasses is limited, and this should be better used in combination with other interventions, rather than as a sole universal treatment [45].

Undoubtedly, to efficiently mitigate the risk arising from campylobacters, while at the same time limiting the possibilities for any adaptation and resistance development, it is crucial to know the effectiveness of the antimicrobials used each time, together with their minimal effective doses. Therefore, this study aimed to investigate the potential of three well-known and aforementioned antimicrobial agents (BAC, ERY, and LA) against 12 selected *Campylobacter* spp. raw chicken meat isolates (including seven *C. jejuni* and five *C. coli*), grown in vitro in either planktonic or biofilm monocultures. Additionally, the inhibitory effect of LA on four mixed-culture biofilms, each one composed of three different *Campylobacter* isolates, was also determined; this is due to the fact that the intercellular interactions that may be encountered within mixed-culture biofilms can significantly influence each member isolate’s tolerance and resistance to antimicrobial treatments [46]. For all those experiments, different growth media were used to support bacterial growth, based on preliminary observations. Overall, this study sought to provide useful information on the comparative effectiveness of the three studied antimicrobial agents for the control of *Campylobacter* spp. under various growth modes (i.e., planktonic, biofilm, monocultures, mixed cultures) that could potentially be encountered in food production and clinical settings.

## 2. Results

### 2.1. The Determination of the MICs, MBCs, and MBICs of Antimicrobial Agents against Campylobacter Cultures

Table 1 presents the values of the MICs, MBCs, and MBICs of the three antimicrobial agents (BAC, ERY, and LA) against the *Campylobacter* cultures (planktonic monocultures, biofilm monocultures, and biofilm mixed cultures), grown in the three different media: Mueller–Hinton (MH), MH with 5% *v*/*v* laked horse blood (HB) broths for planktonic cultures, and MH broth with 5% *v*/*v* chicken juice (CJ) broth for biofilm cultures.

Concerning BAC, its MICs/MBCs against the 13 tested *Campylobacter* isolates (including the 12 raw chicken meat isolates and the outbreak derived ATCC 33291 strain) ranged from 0.5 to 8 μg/mL when the planktonic bacterial growth was in a pure MH broth without any blood supplementation. On the other hand, when the bacteria were grown in the MH-HB broth, MICs and MBCs ranged from 1 to 16 μg/mL and from 1 to 32 μg/mL, respectively. It is worth noting that for one *C. coli* isolate (CAMP_097), the MIC/MBC that were recorded in the MH-HB broth were eightfold higher when compared to those recorded in the MH broth (8 and 1 μg/mL, respectively), indicating the protective role of blood supplementation on the tolerance of its cells towards BAC. Considering the eight tested biofilm-forming isolates (CAMP_005/022/025/048/083/091/114/130), the MBICs recorded for the BAC ranged from 1 to 16 μg/mL, indicating the variability in the efficiency of this disinfectant to inhibit biofilm formation by campylobacters, depending on the isolate and its inherent (genotypic) characteristics.

Concerning ERY, the MICs and MBCs varied from 0.5 to 4 µg/mL and from 0.5 to 16 µg/mL, respectively, in the MH broth for nine out of the twelve *Campylobacter* isolates and the ATCC 33291 strain. Interestingly, three isolates (two *C. jejuni* and one *C. coli*) exhibited high-level resistance (HLR) to ERY when their growth was done in MH without blood, with MIC/MBC values ranging from 256 to 1024 μg/mL. However, this was not reflected in the results obtained when their growth was done in the MH-HB broth, where the MICs and MBCs did not exceed 32 μg/mL or 128 µg/mL, respectively. Thus, in this case, the presence of blood seems to increase the isolates’ susceptibility to the antibiotic. It is noteworthy that these three ERY-resistant isolates (CAMP_074/083/091) were among those exhibiting the highest MIC/MBC values for BAC, recorded for both growth media. The MBICs that were recorded for ERY ranged from 0.25 to 32 μg/mL for the eight tested biofilm-forming *Campylobacter* isolates (CAMP_005/022/025/048/083/091/114/130). It is worth noting that the highest MBIC value (i.e., 32 μg/mL) was recorded against those isolates also exhibiting HLR to the antibiotic when tested planktonically.

Like the case of BAC treatments, the presence of blood in the growth medium (i.e., MH-HB broth) also decreased the planktonic susceptibility in ten of the thirteen tested *Campylobacter* isolates (including the ATCC 33291 strain) against LA, with MICs/MBCs ranging from 1024 to 2048 µg/mL (for both growth media). Similarly, the MBICs that were recorded in MH-CJ broth were also in this range (against the eight tested biofilm-forming isolates; CAMP_005/022/025/048/083/091/114/130). However, when the *Campylobacter* isolates were left to grow under mixed-culture conditions (by forming four consortia, each composed of three different isolates), the MBIC values always increased to 4096 µg/mL, indicating a significant decrease in the efficiency of LA to inhibit the formation of those mixed-culture biofilms when compared to monocultures.

**Figure 1:**
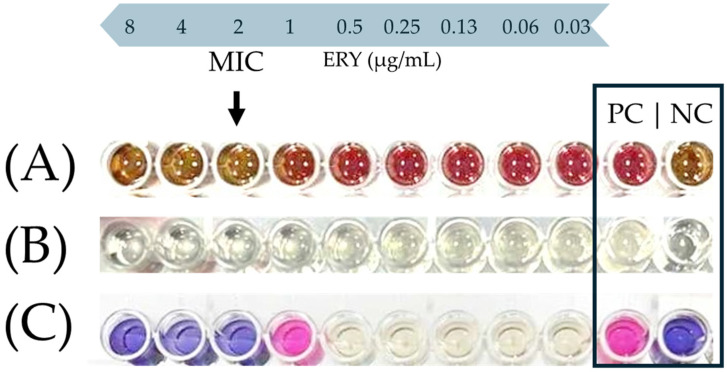
Representative example of MIC endpoint (black arrow) of ERY against the *C. coli* CAMP_005 isolate in the MH-HB broth (**A**), or the MH broth before (**B**) and after the addition of resazurin (**C**). In the last row (**C**), resazurin was added only to the four wells on the left side and the two controls (positive and negative; PC and NC, respectively). This is because there was no reason to also add the dye to the intermediate five wells, since the bacterial growth in them was quite clear to the naked eye due to the increased turbidity of the broth.

Figure 1 shows a characteristic photograph of the well series that was used to determine the MIC of ERY against the *C. coli* CAMP_005 isolate upon its growth in either the MH-HB broth (Figure 1A) or the MH broth (Figure 1B,C). In the latter medium, resazurin sodium salt (Alfa Aesar, Thermo Fisher Scientific Inc., Waltham, MA, USA) was also added in the wells, following the 48 h incubation at 42 °C under microaerophilic conditions as an additional indication of active cellular metabolic activity (Figure 1C). This redox-sensitive dye has a blue color when in its oxidized form, which is converted to pink upon its bioreduction by the living cells [47]. On the other hand, in the case of the MH-HB broth, the inhibition of bacterial growth was solely and empirically verified by the naked eye by observing a change in color of the medium from red to brown (in comparison and similarity to the negative control), since the presence of blood hindered any other absorbance measurement or use of the resazurin dye (Figure 1A).

### 2.2. The Inhibitory Effect of LA against the Biofilm and Planktonic Growth of Each Member Isolate of the Mixed-Culture Campylobacter Consortia

**Figure 2:**
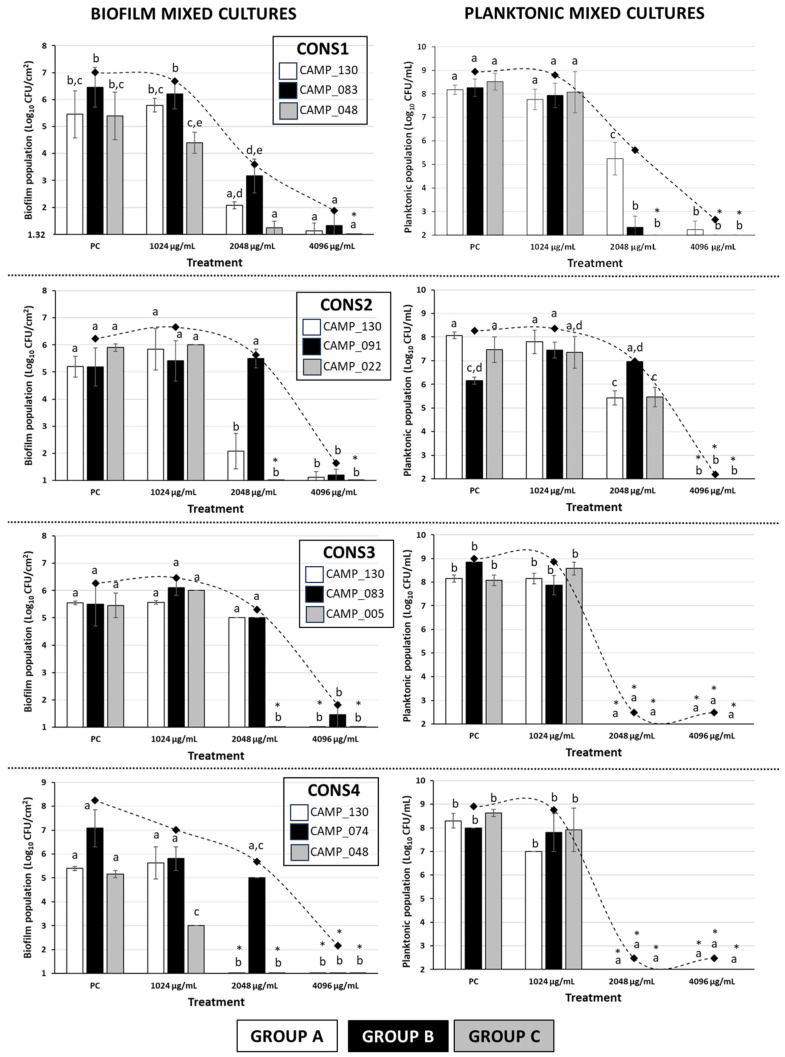
Biofilm and planktonic logarithmic populations (Log_10_ CFU/cm^2^ and Log_10_ CFU/mL, respectively) of each individual *Campylobacter* isolate (n = 7) of the four mixed-culture consortia (i.e., CONS1, CONS2, CONS3, and CONS4), following the 48 h incubation at 42 °C under microaerophilic conditions in the presence of the three tested LA concentrations (i.e., 1024, 2048, and 4096 μg/mL; corresponding to the initial broth pH values of 4.47 ± 0.02, 3.87 ± 0.01, and 3.43 ± 0.03, respectively). In the case of the positive control (PC), the growth was done in the MH-CJ broth without LA addition (initial pH value: 6.76 ± 0.02). Each bar represents the mean values ± standard deviations. The total biofilm and planktonic logarithmic populations for each treatment are also shown as rhombuses, connected by the dotted curved lines. The bars of the standard deviations of those total population means were omitted for clarity. In each graph, the mean values followed by different superscript letters (abcde) differ significantly (*p* < 0.05). Asterisks (*) denote population counts below the detection limits of the plate counting methods (1.32 Log_10_ CFU/cm^2^ and 2 Log_10_ CFU/mL, respectively).

**Figure 3:**
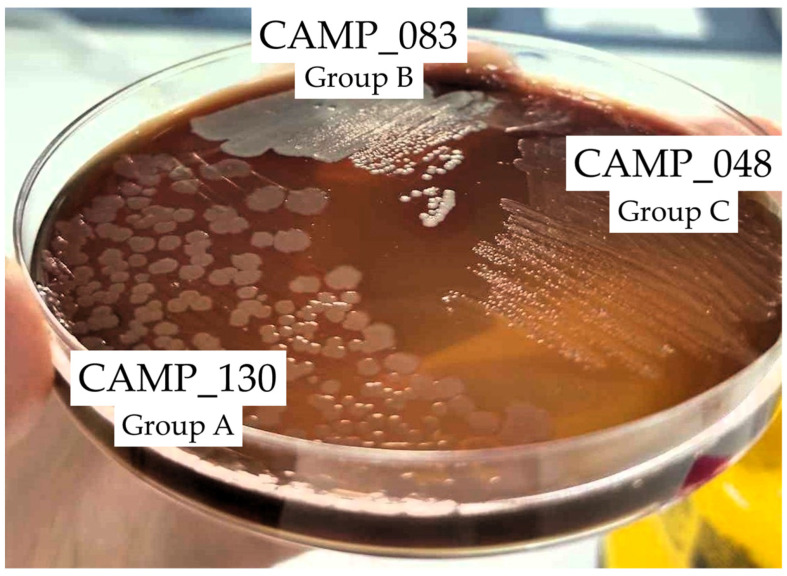
Representative photo of the macroscopic specific characteristics of the colonies of the three *Campylobacter* isolates CAMP_130, CAMP_083, and CAMP_048 that formed the first consortium (CONS1) on the MH-HB agar after 48 h of incubation at 42 °C under microaerophilic conditions.

Figure 2 shows the biofilm and planktonic logarithmic populations (log_10_ CFU/cm^2^ and log_10_ CFU/mL, respectively) for each individual *Campylobacter* isolate (n = 7) of the four mixed-culture consortia (i.e., CONS1, CONS2, CONS3, and CONS4; Table 2), following the 48 h incubation at 42 °C under microaerophilic conditions in the presence of the three tested LA concentrations (i.e., 1024, 2048, and 4096 μg/mL). The discrimination of the seven isolates that formed these consortia was based on their different macroscopic colony characteristics upon their plating on the MH-HB agar. Thus, the isolates in each group (one isolate in Group A and three different isolates in Groups B and C) presented consistent macroscopic colony characteristics on the medium (upon incubation for 48 h at 42 °C under microaerophilic conditions), which varied between groups (Figure 3).

For all four consortia, a significant inhibition of biofilm production occurred when LA was applied at its maximum tested concentration (i.e., 4096 μg/mL). Thus, in this case, an approximately five-log difference when compared to the positive control (PC) was always observed. It is worth noting that the three isolates that were found to present HLR to ERY (i.e., CAMP_74, CAMP_83, and CAMP_91; Group B; Table 1 and Table 2) were also found to exhibit a higher tolerance to LA when this was applied at 2048 µg/mL, compared to most of the other five isolates that belonged to the other two groups (A and C); this also depended on the consortium composition. Interestingly enough, these three isolates had not previously demonstrated strong biofilm formation when tested individually under monoculture conditions (preliminary experiments; data not presented) Additionally, one of those isolates (CAMP_074) was unable to form a biofilm at all when grown individually. Furthermore, in the case of CONS3, despite LA being applied at 2048 µg/mL, a concentration equal to the MBIC against both CAMP_130 and CAMP_083 when grown in monoculture biofilms, the biofilm populations of these isolates were reduced by less than half a log when compared to their positive controls (Table 1). On the other hand, under the mixed-culture conditions, biofilm formation by the three isolates of Group C (i.e., CAMP_005/022/048), which were all strong biofilm formers (when grown individually, and in the absence of any antimicrobial; preliminary experiments; data not presented), appeared to be inhibited by LA more than the other four isolates in most cases. Obviously, all those latter observations denote a strong influence of intercellular interactions that are encountered within mixed-culture *Campylobacter* biofilms on both the population dynamics and the tolerance of each consortium member upon LA exposure.

Regarding the planktonic populations of each consortium member (Figure 2), in two of the consortia (i.e., CONS3 and CONS4), complete growth inhibition was observed (with the final populations being always below the detection limit of the plate counting method; 10^2^ CFU/mL) when LA was applied at a concentration of at least 2048 μg/mL. In these cases, more than six log reductions were observed compared to the positive controls. It is worth noting that, at the same time, biofilm populations exceeding 5 log_10_ CFU/cm^2^ were observed for those two consortia when LA was applied in 2048 μg/mL, a concentration that was fully capable of arresting planktonic growth. On the other hand, the application of the acid at that same concentration against the planktonic growth of the CONS1 members provoked full growth arrest only for isolates CAMP_083 (Group B) and CAMP_048 (Group C), while the isolate CAMP_130 (Group A) was inhibited by approximately three logs. In CONS2, the planktonic cultures of all three member isolates were completely inhibited when LA was applied at its maximum tested concentration (i.e., 4096 μg/mL), which also resulted in the complete inhibition of biofilm formation. For that consortium, when the acid was applied in 2048 μg/mL, an inhibition of the planktonic populations by approximately two and three logs was observed for the isolates of Groups C (CAMP_022) and A (CAMP_130), respectively. On the other hand, the planktonic population of isolate CAMP_091 (Group B) did not show any significant differentiation when compared to the positive control.

Overall, like in the case of biofilm populations, the findings of the selective enumeration of the planktonic populations of each isolate of the four mixed-culture *Campylobacter* consortia also reveal a significant influence of intercellular interactions on the population dynamics and tolerance of each consortium member upon acid exposure. However, differentiations in the behavior of a given isolate may be observed, depending on whether the growth is done under either planktonic or biofilm conditions.

## 3. Discussion

The inhibitory and bactericidal effects of three well-known antimicrobial agents (i.e., BAC, ERY, and LA), all belonging to different classes (i.e., biocide, antibiotic, and natural organic acid, respectively), were initially assessed against the planktonic populations of 12 selected raw chicken meat (wild type) *Campylobacter* isolates in this study. An outbreak derived *C. jejuni* strain (ATCC 33291) was also included in our experiments for comparative purposes. For this, the MICs and MBCs were determined upon growing the planktonic (free-swimming) bacteria in a standard laboratory broth (i.e., MH broth) with or without blood supplementation (5% *v*/*v*) for 48 h at 42 °C under microaerophilic conditions (recommended as the optimal for these bacteria) in both cases. The MIC and MBC results showed a great variability between the isolates, also depending on the growth medium used. Specifically, the MIC and MBC values for both BAC and LA were generally lower when the bacterial growth was done in the blood-free MH broth. Conversely, for ERY, an opposite effect was observed; an increase in the susceptibility of campylobacteria to the antibiotic when these grow in the presence of blood was observed. It is known that *Campylobacter* spp. are fastidious in their growth requirements [48], while it has also been reported that the main species of that genus (i.e., *C. jejuni*) can acquire iron from various sources present in humans, which is critical in establishing infection [49,50]. Interestingly, essential components of blood, namely hemin, hemoglobin, hemin-hemopexin, and hemoglobin-haptoglobin, have been shown to stimulate the growth of *C. jejuni* strains in low-iron media [49]. In accordance with the latter observation, the results of the present study confirmed the stimulating effect blood has on *Campylobacter* planktonic growth, since, in general, most of the tested *C. jejuni* and *C. coli* isolates exhibited better tolerance to the two of the three tested antimicrobial agents, namely BAC and LA, when these were grown in the presence of blood. For instance, this was evident for the *C. coli* CAMP_097 isolate, upon its exposure to BAC, where the observed MIC/MBC values in the presence of blood were eightfold higher (8 μg/mL), compared to when the growth was done without any blood supplementation (1 μg/mL). To the best of our knowledge, there is no literature available comparing the antimicrobial potential of any antimicrobial agent against *Campylobacter* spp. upon their growth, with or without blood supplementation.

Nevertheless, our findings on the MIC values for BAC, regardless of the growth medium (ranging from 0.5 to 16 μg/mL), seem to be consistent with the literature. Thus, the MIC values for that biocide against *C. jejuni* and *C. coli* isolates from several sources (e.g., chicken meat, pork chops, swine cecal contents, rectal swabs, feces, litter of broiler chicken houses) have previously been found within the range of 0.016 to over 64 µg/mL [28,34,51,52]. In an older study testing the sensitivity of planktonic campylobacteria to disinfectants via the filtration method, Avrain et al. showed that 1% *v*/*v* BAC (Barquat DM50 formulation) was effective against 34 *Campylobacter* strains, tested after five minutes of exposure [30]. Moreover, in another suspension disinfection test, a BAC concentration of 0.02% *w*/*v* (200 μg/mL) was able to reduce the population of four *C. jejuni* strains by over six logs CFU/mL after only one minute of exposure [53]. Currently, there are no guidelines available that define antimicrobial endpoint susceptibilities for disinfectants, such as BAC, like there are for antibiotics [54]. However, it should be noted that commercial BAC disinfectants usually contain the bioactive compound at a concentration of at least 0.02% *w*/*v* (200 μg/mL), which is significantly higher than the MBC values observed in the present study [29,53].

The protective effect of blood supplementation on the planktonic growth and antimicrobial tolerance of *Campylobacter* spp. bacteria was also evident for LA, since the MIC values for this acid in MH-HB were consistently twice as high (2048 μg/mL) as the corresponding values in MH (1024 μg/mL), with only three exceptions (isolates CAMP_025/048/091). Consistent with the findings of the present study, previous research has reported MIC values for LA against *C. jejuni* and *C. coli* isolates ranging from 256 to 8192 µg/mL, with most of them ranging from 1024 to 2048 µg/mL [55].

As far as it concerns ERY, in the current study, the MIC values of the antibiotic against the planktonic monocultures ranged from 0.5 to 1024 µg/mL and from 0.25 to 32 µg/mL in MH and MH-HB, respectively, for the 13 tested *C. jejuni* and *C. coli* isolates. These results are again consistent with the literature, as the MIC values of that antibiotic against *C. jejuni* and *C. coli* isolates from several sources have been previously found to range from 0.06 to over 512 µg/mL [28,34,51,52]. In a previous study, Shin and Lee isolated 114 *C. coli* from swine intestinal samples, and 80 of these (that is 70.2%) were found to be resistant to ERY (MIC ≥ 4 μg/mL). Of these, 31 isolates had low-level resistance (MIC = 4–16 μg/mL), and 49 isolates had HLR (MIC ≥ 32 μg/mL) [56]. Active efflux is suggested to contribute to the intrinsic resistance of *Campylobacter* to ERY and to the HLR [56,57,58]. Notably, in our study, three *Campylobacter* isolates, of which two *C. jejuni* (CAMP_074 and CAMP_091) and one *C. coli* (CAMP_083) exhibited HLR to ERY when cultivated in the MH broth with MIC and MBC values ranging from 256 to 1024 µg/mL against those. On the other hand, when these grew in MH-HB, the MICs and MBCs were found to range from 4 to 32 µg/mL and from 64 to 128 µg/mL, respectively. Thus, contrary to the protective effect of blood on the susceptibility of campylobacteria to BAC and LA that we observed in this study, blood presence seems to increase the susceptibility of those microaerophilic pathogens to the macrolide antibiotic. Although this may seem like a high-risk generalization, since our experiments were performed exclusively under in vitro conditions, we may venture to say that this is likely an encouraging result, as the presence of blood is closely related to the site of the action of an internal chemotherapeutic agent, such as ERY, under clinical conditions.

Interestingly, it is worth noting that the MIC value for CAMP_74 observed here when its growth was done in the MH-HB broth (i.e., 4 μg/mL), following the execution of the broth microdilution assay, categorizes this isolate as sensitive, based on the clinical breakpoints published by the European Committee on Antimicrobial Susceptibility Testing (EUCAST) [54]. In agreement with this, the isolate was not denoted as ERY resistant in our subsequent description of the tested isolates, which was based on the execution of the disk diffusion susceptibility test (Kirby–Bauer method) (Section 4.3; preliminary experiments; data not presented). On the other hand, the MIC value that was observed here for the CAMP_083 isolate in the same medium (i.e., 32 μg/mL) categorizes this isolate as ERY resistant, whereas the zone of inhibition we had observed in preliminary experiments (data not presented) during the execution of the Kirby–Bauer method for that isolate and antibiotic did not categorize it as resistant, based again on the EUCAST breakpoints. Nevertheless, ERY was found to present a very-high MIC value against the latter isolate (32 μg/mL when its growth was done in the MH-HB broth), something that surely denotes its HLR to ERY. Differences in these two methods of assessing antimicrobial activity (i.e., disk diffusion susceptibility and broth microdilution tests) should account for these huge discrepancies. It is thus clear that the susceptibility of *Campylobacter* isolates towards ERY (and probably other antibiotics and biocides) appears to be significantly affected by both the presence of blood in the growth medium, as well as the testing method. In addition, it is important to note that the selection of these two isolates (i.e., CAMP_074 (*C. jejuni*) and CAMP_083 (*C. coli*)) for their inclusion in the mixed-culture biofilm consortia of the current study (Table 2) was also based on their particular macroscopic characteristics of their colonies upon their growth on the MH-HB agar, specifically their intense white color (assessed as an atypical colony feature). Overall, the data presented above surely highlight the significance of the growth medium and method in accurately assessing the antimicrobial ability of a chemical agent, particularly in the case of antibiotics.

Following the MIC/MBC determination, the inhibitory effects of the three tested antimicrobial agents (i.e., BAC, ERY, and LA) were evaluated against the formation of monoculture biofilms by the eight tested *Campylobacter* raw chicken meat isolates with biofilm-forming capacity (CAMP_005/022/025/048/083/091/114/130). The other four isolates (CAMP_071/074/097/132) and the ATCC 33291 strain could not form monoculture biofilms (preliminary experiments; data not presented), and were thus excluded from these biofilm susceptibility experiments. In all cases, the MH broth supplemented with 5% *v*/*v* chicken juice (i.e., MH-CJ) was used to support biofilm growth by campylobacters, based on some preliminary findings testing various growth media for their ability to maximize biofilm growth by those bacteria (data not presented). This was an attempt to imitate some of the nutrient conditions that could likely be found in slaughterhouse environments and the poultry industry more generally. In addition, according to some previous authors, CJ has been proposed to enhance the attachment of *C. jejuni* to abiotic surfaces by forming a conditioning film [59,60,61]. For example, the addition of chicken meat exudate to the brucella broth has previously been found to increase biofilm formation by *C. jejuni* on glass, polystyrene (PS), and stainless steel [61]. In this work, the MBIC values for the *Campylobacter* monocultures were almost always equal to or, in some of the cases (isolates), lower than the respective MIC values that were recorded for each antimicrobial agent. Although different growth media were used to support these experiments, this latter observation suggests that the inhibition of planktonic growth should mainly account for the blockage of biofilm formation (i.e., bacteria that are unable to multiply upon planktonic growth may be at a disadvantage to be able to form biofilm). However, in these cases where MBIC values were higher than the respective MIC values (see for instance the cases of the mixed-culture biofilm consortia presented in Figure 2), an additional biofilm-specific inhibitory mechanism (e.g., inhibition of matrix formation, cell-to-cell aggregation, quorum sensing attenuation, etc.) seems to be also involved. This means that, although the planktonic cells are probably unable to multiply, an increasing antimicrobial agent concentration is still required to inhibit biofilm formation by the or part of the mixed-culture population upon its attachment to the surface.

Currently, and to the best of our knowledge, there is no literature available on the inhibitory effect of any of the three tested antimicrobial agents against biofilm formation by *Campylobacter*. However, there is some literature available on the anti-biofilm action of disinfectants (including QACs such as BAC), antibiotics (e.g., ciprofloxacin, erythromycin, tetracycline, meropenem and colistin), and organic acids (other than LA) on pre-formed *Campylobacter* biofilms (either mono- or mixed-cultures with other bacterial species) [31,33,62]. For instance, Rossi et al. conducted a comparative investigation into the effects of five different classes of antibiotics on the planktonic and biofilm forms of 35 strains of *C. jejuni*. This study emphasized how all strains (100%) in biofilms were resistant to erythromycin, meropenem, and colistin, indicating a significant increase in the number of resistant strains when compared to those tested in planktonic form [62]. Intriguingly, in another recent study, a different research team evaluated the synergistic effect of antibiotics and essential oils (EOs) on *Campylobacter* biofilms. They discovered that EOs extracted from *Lavandula stoechas* and *Origanum compactum*, when combined with tetracycline or ampicillin, exhibited a high level of synergy. As a result, a significant reduction of the effective doses of these EOs and antibiotics was observed [63].

Lactic acid (LA) is a valuable bio-product that has gained attention for its various applications. It is commonly used as a food supplement (E270) and an ingredient in cosmetics and hygiene products, as well as for washing carcasses [40,64]. Today, most LA is produced through microbial fermentation, since it can be easily recovered from the fermentation supernatant via electrodialysis, membrane separation, or esterification after removing cells and residual precipitates [65,66,67]. Therefore, LA appears to be an attractive anti-biofilm agent to study. It is also well-recognized that biofilms in the food industry are often composed of mixed species, and these are usually more resistant to disinfectants and antimicrobials than the single-species biofilms often studied under laboratory conditions [68]. Notably, in this work, the MBIC values of LA against all four examined *Campylobacter* consortia were always higher (4096 μg/mL) than the MBIC values recorded for the acid against the corresponding monocultures (1024–2048 μg/mL). This denotes the increased tolerance of mixed cultures to form biofilms compared to monocultures in the presence of that tested organic acid. In a previous mixed-culture biofilm study, *C. jejuni* isolated from retail food samples was found to form more biofilm when co-cultured with *E. coli* or *Pseudomonas aeruginosa* than in a pure culture [69]. Similarly, Teh et al. investigated the attachment of three *C. jejuni* strains to abiotic surfaces, including stainless steel, glass, and PS, both alone and in the presence of *P. aeruginosa* biofilms. The presence of *P. aeruginosa* favored the attachment of two of the three studied strains, while one strain showed better adherence in the monoculture [70]. All the above findings suggest that the bacterial interactions that are encountered within mixed culture consortia can not only influence the attachment ability of campylobacters, but can also influence their biofilm-forming dynamics, along with their AMR and tolerance. Indeed, these significant effects of intercellular interactions on the biofilm-forming dynamics of each *Campylobacter* isolate were also evident in our study upon examining the individual contribution of each *Campylobacter* in the synthesis of the four different consortia exposed to 2048 μg/mL of LA (=1/2 MBIC_consortium_). Thus, depending on the synthesis of each consortium, different isolates were found to dominate in the sessile population, irrespectively of the planktonic population composition.

## 4. Materials and Methods

### 4.1. Antimicrobial Agents (Chemicals) and Preparation of Their Stock Solutions

Benzalkonium chloride (liquid, alkyl distribution from C_8_H_17_ to C_16_H_33_, density: 0.98 g/mL) was purchased from Acros Organics (product code: 215411000; Thermo Fisher Scientific Inc.). ERY was provided by AppliChem GmbH (Erythromycin base BioChemica, product code: A2275,0005; ITW Reagents Division, Darmstadt, Germany), while LA was acquired from PENTA Chemicals Unlimited (L(+)-lactic acid 80%, density: 1.2 g/mL, CAS: 79-33-4; PENTA s.r.o., Prague, Czech Republic). For the preparation of the stock solutions, BAC was dissolved in sterile distilled water (dH_2_O) at a concentration of 10 mg/mL (1% *v*/*v*), ERY was dissolved in absolute ethanol at a concentration of 50 mg/mL (5% *w*/*v*), and LA was dissolved in dH_2_O at a concentration of 163.8 mg/mL (13.7% *v*/*v*). Once prepared, all stock solutions were aseptically filtered through microbiological filters (pore diameter 0.22 µm; Labbox Labware S.L., Barcelona, Spain), and then stored at −20 °C.

### 4.2. The Preparation of Sterile Chicken Juice (CJ)

Minced raw chicken meat (≈300 g) was purchased from a local butcher shop, and then immediately transported to the laboratory. In a plastic stomacher bag, 250 g of the meat were weighed, and 250 mL of sterile dH_2_O were then added (preparing this way a 1:1 dilution). The resulting mixture was thoroughly homogenized in a stomacher (BagMixer^®^ 400; Interscience, Saint Nom la Bretêche, France) for 3 min, then aliquoted into 50 mL plastic Falcon tubes and centrifuged at 7000× *g* for 12 min at 4 °C using a Frontier 5000 Series Multi Pro centrifuge (FC5718R, OHAUS Europe GmbH, Nänikon, Switzerland), in order to remove animal tissue sediment. At the end of centrifugation, the supernatants were carefully removed from each tube and placed into a glass beaker. This aqueous mixture was initially filtered through paper filters (200 g/m^2^; Munktell Filter AB, Falun, Sweden) using a Buchner funnel in order to remove the largest aggregates. The resulting filtrate was then further aseptically filtered through microbiological filters (pore diameter 0.22 µm; Labbox Labware S.L.) and stored at −80 °C.

### 4.3. Campylobacter Isolates and the Preparation of Their Working Cultures

**Figure 4:**
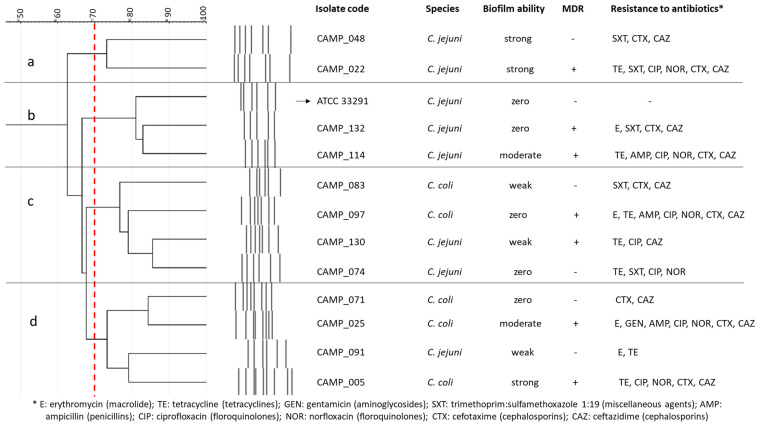
Cluster analysis (dendrogram) based on the rep-PCR genotypic patterns of selected *C. jejuni* (n = 7) and *C. coli* (n = 5) raw chicken meat isolates. Each separate group (a, b, c, d) includes isolates with coefficient similarity of above 70%. The biofilm-forming ability and antibiotic resistance profiles are also presented for each isolate. The *C. jejuni* ATCC 33291 strain is indicated with the arrow.

Seven *C. jejuni* and five *C. coli* isolates, all obtained from raw chicken meat [71], were employed in this study, together with the *C. jejuni* ATCC 33291 strain (a human outbreak isolate) (Figure 4). Their selection was performed to represent isolates with various rep-PCR genotypic patterns, biofilm-forming abilities (i.e., zero, weak, moderate, and strong; categorization based on the crystal violet (CV) staining method for biofilm quantification in microtiter plates proposed by Stepanović et al. [72]), and antibiotic resistance profiles, based on some preliminary results (data not presented). In addition, seven of these isolates were multidrug resistant (MDR), presenting resistance to the antibiotics of at least three different classes. All isolates were kept frozen at −80 °C in MH broth (Oxoid Limited, Thermo Fisher Scientific Inc.) supplemented with 5% *v*/*v* HB (Thermo Fisher Scientific Inc.) and 20% *v*/*v* glycerol (Merck KGaA, Darmstadt, Germany).

For the preparation of the bacterial working cultures, each isolate was initially streaked on the surface of the MH agar (Labbox Labware S.L.) supplemented with 5% *v*/*v* HB and incubated at 42 °C for 24 h under microaerophilic conditions (6.2–13.2% O_2_, 2.5–9.5% CO_2_; Oxoid CampyGen 2.5L Sachet; Thermo Fisher Scientific Inc.) (primary precultures). Secondary precultures were prepared by inoculating a biomass of 5 to 10 colonies from each primary preculture into 2 mL of the fresh MH-HB broth, and then incubating at 42 °C for 24 h under microaerophilic conditions. Working cultures were prepared by transferring 200 μL of each secondary preculture to 1800 μL of fresh MH-HB broth, and then incubating at 42 °C for 24 h under microaerophilic conditions (thereby achieving a final concentration of ca. 10^8^ CFU/mL). In the case of mixed-culture (consortia) experiments (Section 4.6), the final working culture of each isolate was prepared in the MH-CJ broth, independently of the other isolates. Following the growth, the working cultures of the three different isolates that were included in each consortium (Table 2) were combined to achieve the same initial concentration for each one (≈10^5^ CFU/mL), and then left to form biofilms under mixed-culture conditions that are subsequently described (Section 4.6).

### 4.4. The Determination of the MICs and MBCs of Antimicrobial Agents against Campylobacter Planktonic Monocultures

The MIC of each antimicrobial agent (BAC, ERY, and LA) against the planktonic growth of each *Campylobacter* isolate (n = 13) was determined using the broth microdilution method as previously described [73], with some slight modifications made considering the EUCAST reading guide for said method [74]. Briefly, bacteria from each final working culture were inoculated at a starting concentration of ca. 5 × 10^5^ CFU/mL in the MH broth, with or without 5% *v*/*v* HB, and then statically incubated at 42 °C for 48 h under microaerophilic conditions. Eleven different BAC concentrations ranging from 32 to 0.03 μg/mL, sixteen different ERY concentrations ranging from 1024 to 0.03 μg/mL, and seven different LA concentrations ranging from 4096 to 64 μg/mL were tested for each broth. For all three antimicrobial agents, their aforementioned working concentrations were prepared on the day of the experiments via the use of two-fold dilutions of their stock solutions in dH_2_O. Their MICs were finally determined as their lowest concentrations resulting in no visible (by the naked eye) bacterial growth. In the case of the MH broth, this absence of growth was further confirmed through the lack of an increase in the absorbance of the medium measured at 600 nm using a multimode microplate reader (Tecan Spark^®^, Tecan Group Ltd., Männedorf, Switzerland). For this medium, resazurin sodium salt was also used as an additional indicator of metabolic activity [47,75]. For this, the resazurin dye was added in each well at a concentration of 0.01% *w*/*v* following the 48 h incubation, and the potential color change of the metabolized broth (from blue to pink) was observed after another 24 h of incubation at 42 °C.

Following the MIC determinations, to calculate the MBCs, 10 μL of the broth cultures were aspirated from all the non-growth wells of the MIC assays, and spotted (in duplicate) on MH agar plates, which were then incubated at 42 °C for 48 h under microaerophilic conditions. For each bacterial isolate, the MBC of each antimicrobial agent was determined as its lowest concentration which reduced the initial inoculum by more than 99.9% (no appearance of colonies at the point of the spot).

### 4.5. The Determination of the MBICs of Antimicrobial Agents against Campylobacter Monocultures

The MBIC of each antimicrobial agent (BAC, ERY, and LA) against each of the eight *Campylobacter* isolates (CAMP_005/022/025/048/083/091/114/130) that were able to form biofilms under monoculture conditions (preliminary experiments; data not presented) was determined using the CV staining assay as previously described [76]. For this, bacteria were left to form biofilms on 96-well PS microtiter plates (transparent, flat, Cat. No. 30096, SPL Life Sciences Co., Ltd., Naechon-Myeon, Pocheon-si, Gyeonggi-do, Republic of Korea) for 48 h in the MH broth supplemented with 5% *v*/*v* CJ at 42 °C under microaerophilic conditions, in the presence of varying concentrations of each antimicrobial agent, which were the same as those examined for the MIC assay (Section 4.4). The conditions tested (i.e., growth medium, temperature, and the incubation period) have previously been found to maximize biofilm formation by the tested isolates (data not presented). At the end of the incubation, the accumulated biofilm biomass in each well was quantified following its staining with CV (0.1% *w*/*v*), the solubilization of the bound dye with an ethanol/acetone mixture (80:20, *v*/*v*), and the absorbance measurements of the resulting solution at 590 nm (A_590nm_) using the Tecan Spark^®^ multimode microplate reader. As a positive biofilm control, wells containing inoculated ΜH-CJ without antimicrobial agent addition were used, whereas wells containing uninoculated MH-CJ were employed for the negative control. For each bacterial isolate, the MBIC of each antimicrobial agent was determined as its lowest concentration that completely inhibited biofilm formation (the biomass accumulated was not significantly different from that of the negative control).

### 4.6. The Determination of the MBICs of LA against Campylobacter Mixed Cultures

The MBICs of LA against four *Campylobacter* mixed cultures (consortia), each composed of three different isolates (Table 2), were determined following the procedure that was previously described for the MBIC determination against the monocultures (Section 4.5). In this case, however, eight different LA concentrations (two-fold dilutions, ranging from 8192 to 64 μg/mL) were tested. The selection of the seven *Campylobacter* isolates that were included in these four consortia was based on three attributes: the MDR character (Group A), HRL to ERY but not MDR (Group B), and strong biofilm production capacity (Group C). Each consortium was then formed to contain isolates representing all these three attributes (groups). It is worth noting that one of the isolates (CAMP_074) that was included in the fourth consortium (CONS4) was unable to form biofilm under monoculture conditions (data not presented). In addition, the isolates that were included in each group presented different macroscopic colony characteristics on the MH-HB agar compared to the isolates of the other groups, something that enabled the ease of the discrimination of each consortium member isolate upon agar plate counting (Section 4.7).

### 4.7. The Selective Quantification of the Planktonic and Biofilm Populations of Each Campylobacter Isolate of Mixed Cultures (Consortia), with or without LA

To determine the selective inhibitory action of LA against each *Campylobacter* consortium member, the populations of both the planktonic and biofilm cells of each isolate (n = 7) were quantified through agar plating at the end of the MBIC assay at the three highest tested concentrations for this agent (i.e., 1024, 2048, and 4096 μg/mL). To do these experiments, at the end of the 48 h incubation period, for each different consortium (n = 4) and treatment, the planktonic populations were collected from two replicate wells (total volume 400 μL), transferred to 1.5 mL Eppendorf tubes, and then mixed thoroughly using a vortexer (VXMNAL, Ohaus Europe GmbH). Subsequently, six serial decimal dilutions were prepared in a quarter-strength Ringer’s solution (Lab M, Heywood, Lancashire, UK), and from each of those dilutions, the MH-HB plates were inoculated (in duplicate) with either 10 μL or 100 μL of the bacterial suspensions (agar spot and spreading methods, respectively; both these methods were used in parallel for repetitive purposes). The inoculated plates were incubated at 42 °C for 48 h under microaerophilic conditions, and the developed colonies for each isolate were then counted in order to determine the planktonic populations (CFU/mL) that existed in the wells at the time of sampling (48 h).

To quantify the biofilm cells of each consortium member, at the end of the 48 h incubation, the planktonic suspensions were totally removed, the wells were washed twice with a ¼ Ringer’s solution (to remove the loosely attached cells), and 200 μL of this latter solution were added into each well. The submerged surface of each well was then thoroughly scratched with a plastic pipette tip, removing the strongly attached biofilm bacteria, which were again quantified (per isolate) by enumerating their discrete colonies on the MH-HB plates. The cellular concentrations of the biofilm-derived suspensions (CFU/mL) were finally converted to CFU/cm^2^, considering the total surface area (cm^2^) of each well that was initially covered by the 200 µL of the ΜH-CJ broth. This area was calculated using the following equation:

π·r(r+2h),

where π is the mathematical constant defined as the ratio of the circumference to the diameter of a circle (approximately equal to 3.14), r is the radius of each well (0.55 cm), and h is the height of the submerged surface of each well (1 cm).

### 4.8. Statistics

Each experiment was repeated three times, starting from independent bacterial cultures. Planktonic and biofilm plate counts (CFU/mL and CFU/cm^2^, respectively) were transformed to logarithms before the means and standard deviations were computed. The derived data on the planktonic and biofilm logarithmic populations (log_10_ CFU/mL and log_10_ CFU/cm^2^, respectively) were then all submitted to the factorial analyses of variance (ANOVA), followed by Tukey’s multiple range post hoc honestly significant difference (HSD) tests for mean comparison, using the statistical software STATISTICA^®^ v12.0 (StatSoft Inc., Tulsa, OK, USA). Significant differences were always reported at a *p* level of <0.05.

## 5. Conclusions and Perspectives

The present findings provide insight into the comparative antimicrobial effectiveness of three commonly used antimicrobial agents: the general-purpose biocide BAC, the macrolide antibiotic ERY, and the natural organic acid LA, against the planktonic and biofilm growth of 12 representative *Campylobacter* foodborne (wild type) isolates, belonging to the two main species responsible for most human infections (i.e., *C. jejuni* and *C. coli*). They also highlight the significant effects of growth mode (i.e., planktonic vs. biofilm), growth media (blood presence), bacterial interactions (i.e., monocultures vs. mixed cultures), and the inherent (genotypic) characteristics of each isolate on both its biofilm-forming dynamics and antimicrobial tolerance. Future research should determine the antimicrobial effectiveness of these and possibly other (already used, and likely other novel and preferably sustainable) agents against mixed-species *Campylobacter* biofilms, formed under some more relevant food-processing conditions (mainly with respect to the incubation temperature). Surely, the in vitro efficiency of any novel chemotherapeutic agent should also be tested under in vivo conditions in order to confirm its capability to fight these pathogenic bacteria inside their human or animal host. The derived knowledge may help to decrease the prevalence of these microaerophilic important pathogens from the food production chain, along with the associated risks for humans.

## Figures and Tables

**Table 1:** The MICs, MBCs, and MBICs of the three antimicrobial agents (BAC, ERY, and LA) against the *Campylobacter* cultures (planktonic monocultures, biofilm monocultures, and biofilm mixed cultures).

Antimicrobial Agent	*Campylobacter*/Consortium Code		MIC ^1^	MBC ^2^	MIC	MBC	MBIC ^3^
		Species	μg/mL				
			in MH ^4^		in MH-HB ^5^		in MH-CJ ^6^
BAC	ATCC 33291	*C. jejuni*	2	4	4	8	nbf ^7^
	CAMP_005	*C. coli*	2	4	8	16	2
	CAMP_022	*C. jejuni*	2	2	4	8	2
	CAMP_025	*C. coli*	2	4	8	16	4
	CAMP_048	*C. jejuni*	2	2	4	8	1
	CAMP_071	*C. coli*	0.5	1	2	4	nbf
	CAMP_074	*C. jejuni*	4	4	16	32	nbf
	CAMP_083	*C. coli*	8	8	16	32	16
	CAMP_091	*C. jejuni*	8	8	16	32	8
	CAMP_097	*C. coli*	1	1	8	8	nbf
	CAMP_114	*C. jejuni*	2	4	1	1	2
	CAMP_130	*C. jejuni*	4	4	8	16	8
	CAMP_132	*C. jejuni*	1	1	4	4	nbf
ERY	ATCC_33291	*C. jejuni*	2	2	1	2	nbf
	CAMP_005	*C. coli*	2	4	2	4	2
	CAMP_022	*C. jejuni*	4	4	1	2	1
	CAMP_025	*C. coli*	4	16	4	8	2
	CAMP_048	*C. jejuni*	1	2	0.5	1	0.5
	CAMP_071	*C. coli*	0.5	0.5	0.25	0.25	nbf
	CAMP_074	*C. jejuni*	256	256	4	64	nbf
	CAMP_083	*C. coli*	1024	1024	32	128	32
	CAMP_091	*C. jejuni*	1024	1024	16	64	32
	CAMP_097	*C. coli*	2	4	1	2	nbf
	CAMP_114	*C. jejuni*	4	4	0.5	1	0.25
	CAMP_130	*C. jejuni*	0.5	0.5	0.25	0.5	0.25
	CAMP_132	*C. jejuni*	2	2	1	1	nbf
LA	ATCC_33291	*C. jejuni*	1024	1024	2048	2048	nbf
	CAMP_005	*C. coli*	1024	1024	2048	2048	1024
	CAMP_022	*C. jejuni*	1024	1024	2048	2048	1024
	CAMP_025	*C. coli*	1024	1024	1024	1024	1024
	CAMP_048	*C. jejuni*	2048	2048	2048	2048	1024
	CAMP_071	*C. coli*	1024	1024	2048	2048	nbf
	CAMP_074	*C. jejuni*	1024	1024	2048	2048	nbf
	CAMP_083	*C. coli*	1024	2048	2048	2048	2048
	CAMP_091	*C. jejuni*	2048	2048	2048	2048	1024
	CAMP_097	*C. coli*	1024	1024	2048	2048	nbf
	CAMP_114	*C. jejuni*	1024	1024	2048	2048	1024
	CAMP_130	*C. jejuni*	1024	1024	2048	2048	2048
	CAMP_132	*C. jejuni*	1024	1024	2048	2048	nbf
	CONS1 (CAMP_048/083/130)	*C. jejuni*/*C. coli*/*C. jejuni*	nd ^8^	nd	nd	nd	4096
	CONS2 (CAMP_022/091/130)	*C. jejuni*/*C. jejuni*/*C. jejuni*	nd	nd	nd	nd	4096
	CONS3 (CAMP_005/083/130)	*C. jejuni*/*C. coli*/*C. jejuni*	nd	nd	nd	nd	4096
	CONS4 (CAMP_048/074/130)	*C. jejuni*/*C. jejuni*/*C. jejuni*	nd	nd	nd	nd	4096

^1^ Minimum Inhibitory Concentration; ^2^ Minimum Bactericidal Concentration; ^3^ Minimum Biofilm Inhibitory Concentration; ^4^ Mueller–Hinton broth; ^5^ MH with 5% *v*/*v* laked horse blood (HB); ^6^ MH broth with 5% *v*/*v* chicken juice (CJ); ^7^ non biofilm former; ^8^ not determined.

**Table 2:** The four mixed-culture consortia, each composed of three different *Campylobacter* isolates. The seven isolates that formed these consortia were divided into three different groups (A–C), depending on their drug resistance and biofilm-forming phenotypes.

Consortium Code	Group A ^1^	Group B ^2^	Group C ^3^
CONS1	*C. jejuni* (CAMP_130)	*C. coli* (CAMP_083)	*C. jejuni* (CAMP_048)
CONS2	*C. jejuni* (CAMP_130)	*C. jejuni* (CAMP_091)	*C. jejuni* (CAMP_022)
CONS3	*C. jejuni* (CAMP_130)	*C. coli* (CAMP_083)	*C. jejuni* (CAMP_005)
CONS4	*C. jejuni* (CAMP_130)	*C. jejuni* (CAMP_074)	*C. jejuni* (CAMP_048)

^1^ Multidrug resistance (MDR) (preliminary experiments; data not presented); ^2^ Non MDR, with HLR to ERY (current findings); ^3^ Strong biofilm former (preliminary experiments; data not presented).

## Data Availability

The data presented in this study are contained within the article.
